# Phylogeography and Taxonomy of *Trypanosoma brucei*

**DOI:** 10.1371/journal.pntd.0000961

**Published:** 2011-02-08

**Authors:** Oliver Balmer, Jon S. Beadell, Wendy Gibson, Adalgisa Caccone

**Affiliations:** 1 Department of Medical Parasitology and Infection Biology, Swiss Tropical and Public Health Institute, Basel, Switzerland; 2 Department of Ecology and Evolutionary Biology, Yale University, New Haven, Connecticut, United States of America; 3 Institute of Zoology, University of Basel, Basel, Switzerland; 4 Molecular Systematics and Conservation Genetics Laboratory, Yale Institute for Biospheric Studies, Yale University, New Haven, Connecticut, United States of America; 5 School of Biological Sciences, University of Bristol, Bristol, United Kingdom; IRD/CIRDES, Burkina Faso

## Abstract

**Background:**

Characterizing the evolutionary relationships and population structure of parasites can provide important insights into the epidemiology of human disease.

**Methodology/Principal Findings:**

We examined 142 isolates of *Trypanosoma brucei* from all over sub-Saharan Africa using three distinct classes of genetic markers (kinetoplast CO1 sequence, nuclear SRA gene sequence, eight nuclear microsatellites) to clarify the evolutionary history of *Trypanosoma brucei rhodesiense* (*Tbr*) and *T. b. gambiense* (*Tbg*), the causative agents of human African trypanosomosis (sleeping sickness) in sub-Saharan Africa, and to examine the relationship between *Tbr* and the non-human infective parasite *T. b. brucei* (*Tbb*) in eastern and southern Africa. A Bayesian phylogeny and haplotype network based on CO1 sequences confirmed the taxonomic distinctness of *Tbg* group 1. Limited diversity combined with a wide geographical distribution suggested that this parasite has recently and rapidly colonized hosts across its current range. The more virulent *Tbg* group 2 exhibited diverse origins and was more closely allied with *Tbb* based on COI sequence and microsatellite genotypes. Four of five COI haplotypes obtained from *Tbr* were shared with isolates of *Tbb*, suggesting a close relationship between these taxa. Bayesian clustering of microsatellite genotypes confirmed this relationship and indicated that *Tbr* and *Tbb* isolates were often more closely related to each other than they were to other members of the same subspecies. Among isolates of *Tbr* for which data were available, we detected just two variants of the SRA gene responsible for human infectivity. These variants exhibited distinct geographical ranges, except in Tanzania, where both types co-occurred. Here, isolates possessing distinct SRA types were associated with identical COI haplotypes, but divergent microsatellite signatures.

**Conclusions/Significance:**

Our data provide strong evidence that *Tbr* is only a phenotypic variant of *Tbb*; while relevant from a medical perspective, *Tbr* is not a reproductively isolated taxon. The wide distribution of the SRA gene across diverse trypanosome genetic backgrounds suggests that a large amount of genetic diversity is potentially available with which human-infective trypanosomes may respond to selective forces such as those exerted by drugs.

## Author Summary

*Trypanosoma brucei*, the parasite causing human African trypanosomiasis (sleeping sickness) across sub-Saharan Africa is traditionally split into three subspecies: *T. b. gambiense* (*Tbg*), causing a chronic form of human disease in West and Central Africa; *T. b. rhodesiense* (*Tbr*), causing an acute form of human disease in East and Southern Africa; and *T. b. brucei* (*Tbb*), which is restricted to animals. *Tbg* is further split into *Tbg* group 1 and *Tbg* group 2. Better understanding the evolutionary relationships between these groups may help to shed light on the epidemiology of sleeping sickness. Here, we used three different types of genetic markers to investigate the phylogeographic relationships among the four groups across a large portion of their range. Our results confirm the distinctiveness of *Tbg* group 1 while highlighting the extremely close relationships among the other three taxa. In particular, *Tbg* group 2 was closely related to *Tbb*, while *Tbr* appeared to be a variant of *Tbb*, differing only in its phenotype of human infectivity. The wide geographic distribution of the gene conferring human infectivity (SRA) and the fact that it is readily exchanged among lineages of *T. brucei* in eastern Africa suggests that human-infective trypanosomes have access to an extensive gene pool with which to respond to selective pressures such as drugs.

## Introduction

*Trypanosoma brucei* is a unicellular flagellated parasite restricted to sub-Saharan Africa by the distribution of its tsetse vector (*Glossina* spp.) [1]. It has caused periodically devastating epidemics of human sleeping sickness. In the last decade, the annual number of new cases has decreased [2], [3]; currently, the World Health Organization estimates that among the millions of people at risk across 36 countries, sleeping sickness causes approximately 50,000 deaths each year [4], [5]. However, geographically restricted outbreaks can still cause severe economic and social disruption [6], [7] and past disease cycles suggest that new epidemics could occur at any time [8]. In addition, appropriate drugs to treat the disease are still lacking [9].

Taxonomically, *T. brucei* is divided into three subspecies, largely based on their geographical origin, infectivity to humans and severity of disease. *T. b. gambiense* (*Tbg*) is restricted to West and Central Africa, where it causes a chronic form of sleeping sickness in humans. The Gambian form of sleeping sickness, caused by *Tbg*, was traditionally viewed as primarily a human infection, but it has become clear that a broad range of wild and domestic animal reservoirs also harbor the parasite [10], [11], [12]. A second human-infective subspecies, *T. b. rhodesiense* (*Tbr*), is found in eastern and southern Africa and causes an acute form of sleeping sickness. *Tbr* is a zoonotic disease for which non-human vertebrates are the primary reservoir. The third subspecies, *T. b. brucei* (*Tbb*), is distributed across sub-Saharan Africa, and is restricted to non-human vertebrates, in which it can cause nagana, a chronic wasting disease [13].

Over the last three decades, population genetic research has provided important insights into the biology of *T. brucei* and the epidemiology of sleeping sickness [14], [15], [16], [17], [18], [19], [20], [21], [22], [23], [24]. But the fine scale ecological and evolutionary processes underlying disease dynamics and the distinction of the different parasite forms are still not very well understood. From a taxonomic standpoint, this previous work has clearly established that *Tbg* is genetically distinct from *Tbr* and *Tbb* [14], [15], [24], [25]. However, there is still a debate whether *Tbg* is evolutionarily older than *Tbb*/*Tbr*. As *Tbg* is less virulent than *Tbr*, there is a widespread belief that *Tbg* is evolutionarily older than *Tbb*/*Tbr*, based on the assumption that parasites generally evolve towards becoming more benign as they adapt to their host, an assumption that not supported by evidence [26], [27]. In addition, the evolutionary relationship between *Tbr* and *Tbb* remains enigmatic; they are morphologically indistinguishable, sympatric in large parts of eastern Africa, and differentiated solely by their capacity to infect humans. Epidemics involving *Tbr* tend to occur in more or less discrete foci and may involve multiple *Tbr* lineages [28], [29], , but sometimes, a single lineage of *Tbr* may clonally expand to high frequency. Consequently, when population genetic structure is characterized over a small geographical range or over a small time frame, *Tbr* and *Tbb* may appear deceptively isolated from each other [31], [32]. On the other hand, *Tbr* may evolve through frequent genetic exchange with sympatric *Tbb*, leading to a mosaic of different *Tbr* genotypes distributed throughout endemic regions of eastern Africa [30], [33]. Laboratory studies have demonstrated that *T. brucei* is capable of sexual reproduction [34] and that crosses between *Tbb* and *Tbr* can produce viable progeny [35]. The extent to which this occurs in nature is unknown, but concurrent infections with multiple *T. brucei* genotypes are common [36], providing ample opportunity for genetic exchange. The finding that isolates of *Tbr* from Uganda were more closely related to sympatric *Tbb* than to *Tbr* from Zambia supports the hypothesis that human-infective parasite may have had multiple origins in Africa [37].

Human infectivity in *Tbr* has been attributed to the serum resistance associated (SRA) gene [38], [39]. The SRA gene has been PCR-amplified exclusively from human-infective trypanosome stocks [40] and transfection of *Tbb* with the SRA gene is sufficient to confer resistance to human serum [39]. Therefore, in eastern Africa, the SRA gene has emerged as a useful marker for identifying human-infective trypanosomes in their animal reservoir [33], [40], [41]. Given the potential for recombination between *Tbr* and *Tbb* [35], the SRA gene could potentially occur on all genetic backgrounds (i.e. turning *Tbb* into *Tbr* through recombination). This would imply that the standing genetic variation and associated phenotypic variation present in all *T. brucei* parasites in eastern and southern Africa, could eventually occur in a human-infective form. The questions remain if *T. brucei* lineages exist that are incapable of hosting the SRA gene and if *Tbb* and *Tbr* are simply host-range variants. Insights into these questions will be critical for more clearly defining the pool of parasites responsible for human disease, for understanding the emergence of new disease foci, and for eventually understanding how human-infectivity interacts with the evolution of other important traits such as animal host range [42], parasite fitness [43], virulence [44], [45], [46] and drug-resistance [47].

Our goal in this study was to clarify the evolutionary history of *T. brucei* and to more finely resolve the relationships between *Tbr* and *Tbb* from eastern and southern Africa, explicitly accounting for the SRA status of isolates. To accomplish this, we estimated phylogenetic relationships of all three subspecies using kinetoplast (mitochondrial) DNA sequence and integrated this with estimates of population structure based on nuclear microsatellite variation. We then examined the extent to which the distribution of the two existing lineages of the SRA gene among *Tbr* isolates matched the phylogenetic and population genetic patterns.

## Methods

### Sampling

**Figure 1:**
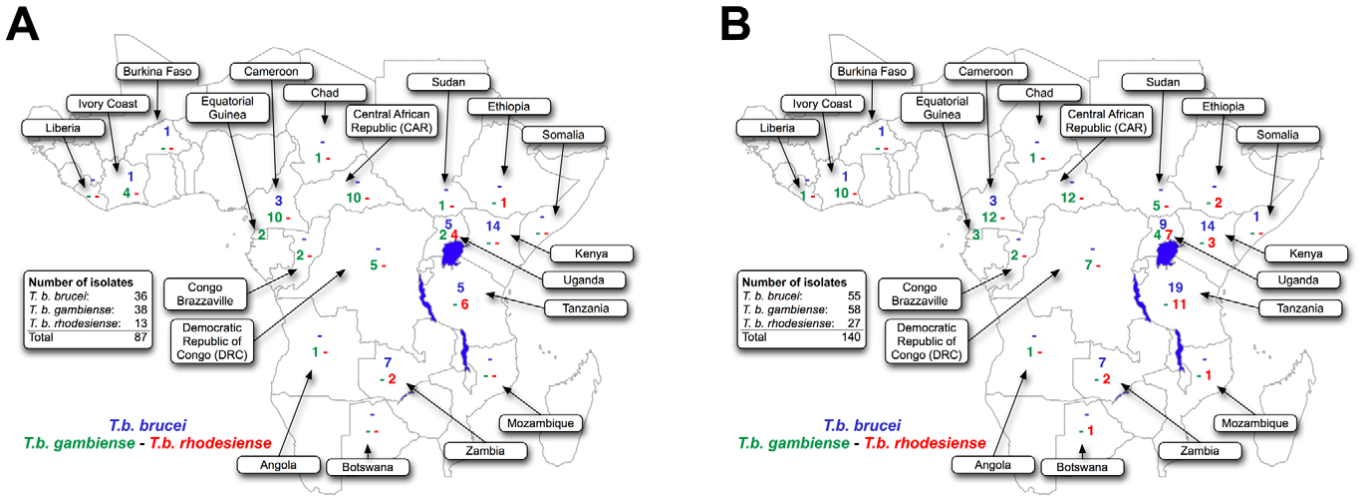
Distribution of 142 *Trypanosoma brucei* isolates used. Geographic origin of A) 87 *Trypanosoma brucei* isolates included in the phylogenetic analysis of partial CO1 sequences and B) 140 *T. brucei* isolates genotyped at 8 microsatellite loci for population genetic analysis. For each country a triplet of numbers or dashes indicates sample sizes for *T. b. brucei* (blue), *T. b. gambiense* (group 1 and group 2 inclusive; green), and *T. b. rhodesiense* (red).

We obtained 142 isolates of *T. b. brucei*, *T. b. rhodesiense* and *T. b. gambiense* live, lysed, or as extracted DNA from the Swiss Tropical Institute Basel (STIB, Reto Brun), University of Bristol (Wendy Gibson), CIRAD-IRD/LRCT, Montpellier (Pascal Grébaut), IRD, Montpellier (Anne Clarisse Lekane), and Yale University (Serap Aksoy) (see supplementary material, Table S1). All isolates had been expanded in mice or in axenic culture in the past. Consequently, the diversity of parasite genotypes occurring in the wild may have been reduced by artificial selection pressures while cultures were maintained in an atypical environment [48]. All isolates were isolated in previous studies in adherence with national and institutional guidelines. Trypanosome isolates from patients were collected in previous studies as part of diagnostic procedures according to local ethical guidelines and were treated anonymously. Of those isolates with known host species, 16 (11%) were originally isolated from tsetse flies, 73 (52%) were from humans, and 52 (37%) from other vertebrate hosts. The geographical origin of these isolates, which spans sub-Saharan Africa, is indicated in Figure 1.

Isolates of *T. b. gambiense* had been previously assigned five different taxonomic labels: *Tbg*, *Tbg* group 1, *Tbg* group 2, *Tbg* “non group 1” and “*Tb* non-*gambiense* group 1”. *Tbg* and *Tbg* group 1 were considered to be synonymous here and are referred to collectively as *Tbg* group 1. This group, which comprises classical *Tbg*, is distinguished from the more virulent and genetically distinct taxon *Tbg* group 2, which was originally found in Ivory Coast [11], [49]. Isolates originally classified as “*Tbg* non group 1” or “*Tb* non-*gambiense* group 1” (ob152–ob155), for which human infectivity has not been established, were treated as *Tbb*.

### Genotyping

Depending on the quality of the material, DNA was extracted using either a DNA extraction kit (Qiagen) or using phenol/chloroform extraction. Partial cytochrome c oxidase subunit I (CO1) was amplified from the kinetoplast (kDNA) genome of a subset of samples using primers Max1 (5′-ccctacaacagcaccaagt) and Max2 (5′-ttcacatgggttgattatgg) designed to the CO1 open reading frame contained in the maxicircle sequence of *T. b. brucei* 427 (GenBank accession no. M94286) and sequenced on an ABI3730 Genetic Analyzer (Applied Biosystems Inc.). Sequences were aligned by eye with Sequencher 4.2 (Gene Codes Corporation, Ann Arbor, MI). All isolates were also typed at eight dinucleotide microsatellite loci (TB1/8, TB2/19, TB5/2, TB6/7, TB8/11, TB9/6, TB10/5, TB11/13) using conditions described previously [50]. These loci, which are located on eight different chromosomes are not physically linked [51]. Isolates exhibiting three or more alleles at any locus were considered to harbor multiple infections [36], [50] and were excluded from this analysis.

### SRA detection

We tested samples identified as *Tbb* and *Tbr* for the presence of the SRA gene. We performed PCR detection using the primers and protocols developed by Gibson et al. [33] (primers SRA A/E) and Radwanska et al. [52] (primers SRA F/R). Products from primers SRA A/E were sequenced on an ABI3730 Genetic Analyzer. As a control to help ensure that failure to amplify SRA using either of these primer sets was not attributable to poor DNA quality, we also tested the same samples for amplification of a single-copy microsatellite (Tb 9/6, [50]). For some isolates we incorporated the results of prior typing efforts [33], [52]. We limited this analysis to *Tbb* and *Tbr* since SRA has not been detected in *Tbg* groups 1 and 2 [33], [52], [53].

### Phylogenetic and phylogeographic analysis of kDNA sequences

A phylogenetic tree of kinetoplast sequences was estimated using the Bayesian approach implemented in MrBayes [54]. Plotting of the appropriate maximum likelihood (ML) distance as determined by Modeltest [55] against the uncorrected p-distance for all sample pairs revealed saturation of the third codon position between ingroup and outgroup. Therefore third codon positions and combined first and second codon positions were treated as two separate partitions. The hierarchical likelihood ratio test implemented in MrModeltest [56] identified the Hasegawa, Kishino and Yano [57] model with gamma (HKY85+G) as the most appropriate nucleotide substitution model for the data in both partitions. Phylogenetic relationships were also estimated using maximum parsimony as implemented in PAUP* [58]. Bootstrap support was estimated using 1000 replicates. Trees were rooted with available sequences of *T. cruzi* (GenBank accession no. DQ343646), *T. vivax*, and *T. congolense* as outgroups (the two latter sequences were produced by the Pathogens Sequencing Group at the Sanger Sequencing Centre and can be obtained from GeneDB.org). We assessed geographical and taxonomic patterns in haplotype distribution using a haplotype network constructed using the statistical parsimony approach implemented in the program TCS 1.21 [59]. Sub-networks were created using the 99% confidence limit settings. Subsequently, sub-networks were connected to each other by relaxing the confidence limit. Divergence between subnetworks was calculated in the program DnaSP [60].

### Analysis of microsatellite variation

We used the individual-based Bayesian clustering approach implemented in the program STRUCTURE [61] to explore the hierarchical genetic relationships among all parasite isolates. For sexually recombining organisms, STRUCTURE estimates the proportion of each individual's genome that is derived from one of *K* pre-specified populations. In the case of an often clonal organism such as *T. brucei*, inferred “populations” are likely to reflect the major clades of the coalescent tree and these clusters can help to describe the structure of genetic variation (J. Pritchard, pers. comm.). To identify the most likely *K*, we conducted 3 independent runs for each K from 1 to 16, assuming an admixture model and correlated allele frequencies. We used a burn-in of 50,000 and replication values of 250,000. We used two methods to determine the most likely number of clusters given the data. In the first, the likelihood values of each *K* (i.e. L(*K*)) were converted into posterior probabilities as suggested by Pritchard et al. [61] to assess which number of subpopulations is most probable given the data. In the second, the greatest value of delta *K*, the second order of change in L(*K*) divided by the standard deviation of L(*K*) was taken as indication for the optimal *K* as suggested by Evanno [62].

We examined whether clusters of genetically similar individuals within the *Tbb/Tbr* group were more similar in geographical origin than expected by chance, given our sampling. For this analysis, individuals were assigned to the single cluster in which they exhibited the highest membership probability. We calculated a statistic that measured the sum of all differences between country of origin (same = 0, different = 1) for all pairwise comparisons among individuals within clusters. We then randomly re-assigned individuals to clusters 1000 times and calculated the same statistic for each permutation. Significance was determined by comparing the observed value to the distribution generated by random permutation. We also performed a similar analysis using date of sampling, but here the statistic was the sum of differences between years of sampling (number of years difference between two isolation events) for all pairwise comparisons among isolates within clusters. Permutations were performed in SAS v 9.1 (SAS Institute, Cary, NC).

We further evaluated the genetic differentiation between subspecies of *T. brucei* using principal components analysis (PCA). This method, which makes no assumptions regarding Hardy-Weinberg or linkage equilibrium, reduces the dimensionality of microsatellite data to two axes, allowing for easy visualization of relative differentiation. PCA was performed in R [63] using the package adegenet [64]. Within subspecies of *T. brucei*, we estimated the differentiation between temporally and geographically cohesive subgroups using D_EST_, an estimate of Jost's D [65] calculated with the program smogd [66]. D_EST_, which varies on a scale from 0 (no differentiation) to 1 (complete differentiation), provides a less biased estimate of differentiation than F_ST_ and related statistics, particularly when estimated using highly polymorphic microsatellite loci [67].

## Results

### Phylogenetic analysis of CO1 sequences

Sequencing of CO1 yielded 812 base pairs with no gaps or stop codons. We recovered a total of 19 distinct haplotypes from the 87 *T. brucei* isolates sequenced (Table S2). These haplotypes exhibited sequence divergence ranging from 0.1% (1 nucleotide substitution) to 4.2% (34 substitutions).

**Figure 2:**
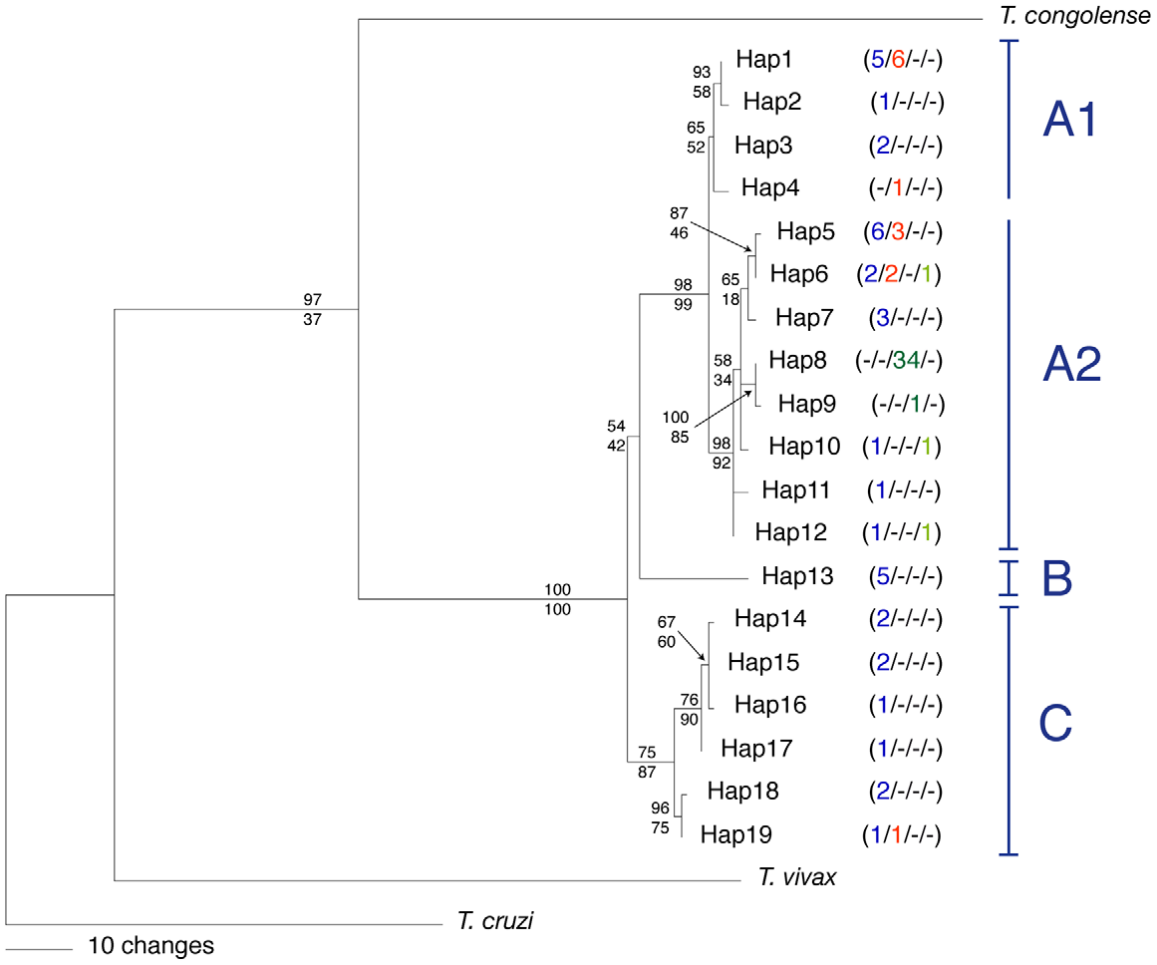
Phylogenetic tree of 87 *Trypanosoma brucei* isolates. 50% majority rule consensus tree from the Bayesian analysis of 812 bp of kDNA (CO1) for *Trypanosoma brucei* and three congeneric outgroups. The frequency with which a particular haplotype was recovered from each of four taxa is indicated in parentheses (left to right: *T. b. brucei* (blue) / *T. b. rhodesiense* (red) / *T. b. gambiense* group 1 (dark green) / *T. b. gambiense* group 2 (light green)). Clade support values for each node are indicated by Bayesian posterior probability (top) and maximum parsimony bootstrap percentage (bottom). *T. b. gambiense* group 1 is represented only by haplotypes Hap8 and Hap9; all other *T. b. gambiense* are group 2. Letters A through C indicate the major clades identified.

With the exception of the placement of Hap13, topologies recovered from Bayesian analysis and from maximum parsimony analysis were almost identical; therefore, we present only the results of the former. The 50% majority rule tree resulting from the Bayesian analysis of kinetoplast haplotypes (Figure 2) revealed one well-differentiated high-level clade (Clade A, Hap1 to Hap 12). Clade A was composed of haplotypes recovered from each of the three subspecies of *T. brucei*, all of which were more closely related to each other than to haplotypes Hap 13 to Hap 19, which formed clades B and C. The latter haplotypes derived from one isolate of *Tbr*, as well as several isolates of *Tbb* that had been previously assigned to the “Sindo” (Hap13) or “Kiboko” groups (Hap14 to Hap19) by kDNA typing [68] or isoenzyme analysis [15] (Table S1).

Within Clade A, haplotypes were further structured, with Clade A2 exhibiting strong Bayesian and bootstrap support. Subclade A2 was composed of all three subspecies of *T. brucei* and contained all haplotypes recovered from *Tbg*. *Tbg* group 1 was represented by only two closely related haplotypes (Hap8 and Hap9), which differed by just one nucleotide. Hap8 was recovered from 34 out of 35 *Tbg* group 1 isolates. Isolates classified as *Tbg* group 2 were represented by three different haplotypes (Hap6, Hap10 and Hap12), each of which was also found in *Tbb* and one of which (Hap6) was also recovered from *Tbr*. A close relationship between *Tbr* and *Tbb* was supported by the fact that four out of the five haplotypes recovered from *Tbr* were also recovered from *Tbb*, and these haplotypes were distributed across the phylogeny.

**Figure 3:**
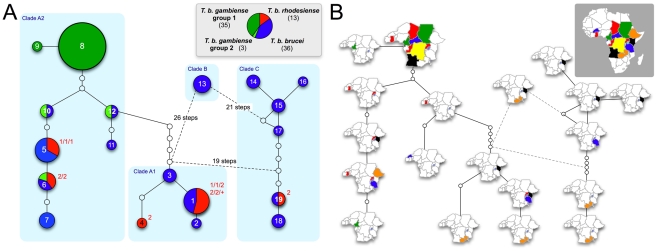
Haplotype network. Maximum parsimony haplotype networks showing genealogical relationships among *Trypanosoma brucei* kinetoplast haplotypes. Panel A highlights the relationships among lineages of *T. b. brucei*, *T. b. rhodesiense*, *T. b. gambiense* group 1 and *T. b. gambiense* group 2 (color-coded). Circles are sized proportional to the frequency with which a particular haplotype was recovered. Numbers in the circles correspond to haplotype ID. Empty circles indicate haplotypes that are inferred to exist but were not sampled. Red numbers next to haplotypes containing *T. b. rhodesiense* indicate the SRA types of the included *T. b. rhodesiense* isolates (1, SRA type 1; 2, SRA type 2; +, SRA type not known). The light blue boxes correspond to the clades defined in Figure 2. Panel B shows the geographic range of each haplotype.

The structure observed in the Bayesian phylogeny was reiterated in a haplotype network (Figure 3A). Haplotype network construction resulted in three separate subnetworks, reflecting the relatively large divergence (∼3%) observed between Clades A, B and C. Clade A was composed of isolates found across all of Africa while Clade C appeared to be restricted to eastern and southern Africa (Figure 3B). Isolates of *Tbb* or *Tbr* from Kenya, Tanzania and Zambia were represented in both Clades A and C. The most commonly recovered haplotype of *Tbg* (Hap8) was found across most of central and western Africa and from every country in which *Tbg* was sampled (Figure 3B).

### Genetic structure - *T. b. brucei* and *T. b. rhodesiense*

**Figure 4:**
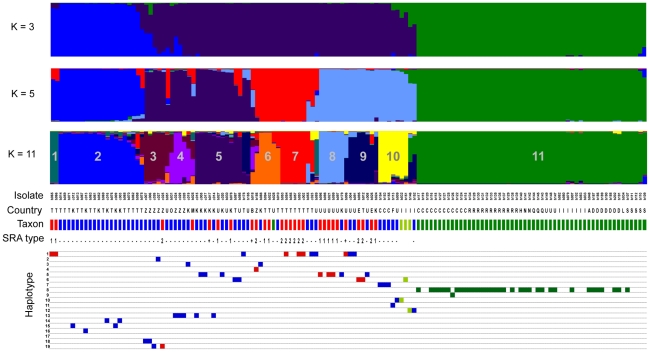
Genetic structure of *Trypanosoma brucei* isolates. Plots show Bayesian clustering of 140 *Trypanosoma brucei* genotypes based on 8 microsatellite loci and their association with kinetoplast haplotypes and the presence or absence of the SRA gene. Clustering of genotypes is shown for K = 3, 5 and 11 partitions (top three panels). The isolate code is indicated for each isolate. The geographical origin of isolates is indicated by a single letter (A, Angola; B, Botswana; C, Cameroon; D, Democratic Republic of Congo; E, Ethiopia; F, Burkina Faso; H, Chad; I, Ivory Coast; K, Kenya; L, Liberia; M, Mozambique; N, Congo Brazzaville; O, Somalia; Q, Equatorial Guinea; R, Central African Republic; S, Sudan; T, Tanzania; U, Uganda; Z, Zambia). The taxonomic assignment of isolates is indicated by color-coded bars across the fourth panel (*T. b. brucei*, blue; *T. b. rhodesiense*, red; *T. b. gambiense* group 1, dark green; *T. b. gambiense* group 2, light green). SRA type is indicated by number when known; otherwise just presence (+) or absence (−) of SRA is indicated. Kinetoplast haplotypes (squares, color coded by taxon), when available, are displayed in the bottom panel.

We used microsatellites to genotype 27 isolates of *Tbr*, 55 isolates of *Tbb* and 58 isolates of *Tbg* collected across Africa (Figure 1B and Table S1). The L(*K*) values derived from STRUCTURE analysis indicated that the probability of our data was maximized by *K* = 11 partitions. Alternatively, ignoring the strong signal derived from the obvious division between parasites identified as *Tbg* group 1 and all other parasites, Evanno's criterion (delta *K*) indicated that our data were most consistent with *K* = 5 partitions. To capture the hierarchical relationships among genotypes, Figure 4 shows the clustering results for *K* = 5 and 11, as well as *K* = 3, corresponding to the number of classically-defined subspecies presumed to be present in our sample. Nesting of clusters (from K = 11 to K = 3) reflects the hierarchical relationships among parasite genotypes.

**Figure 5:**
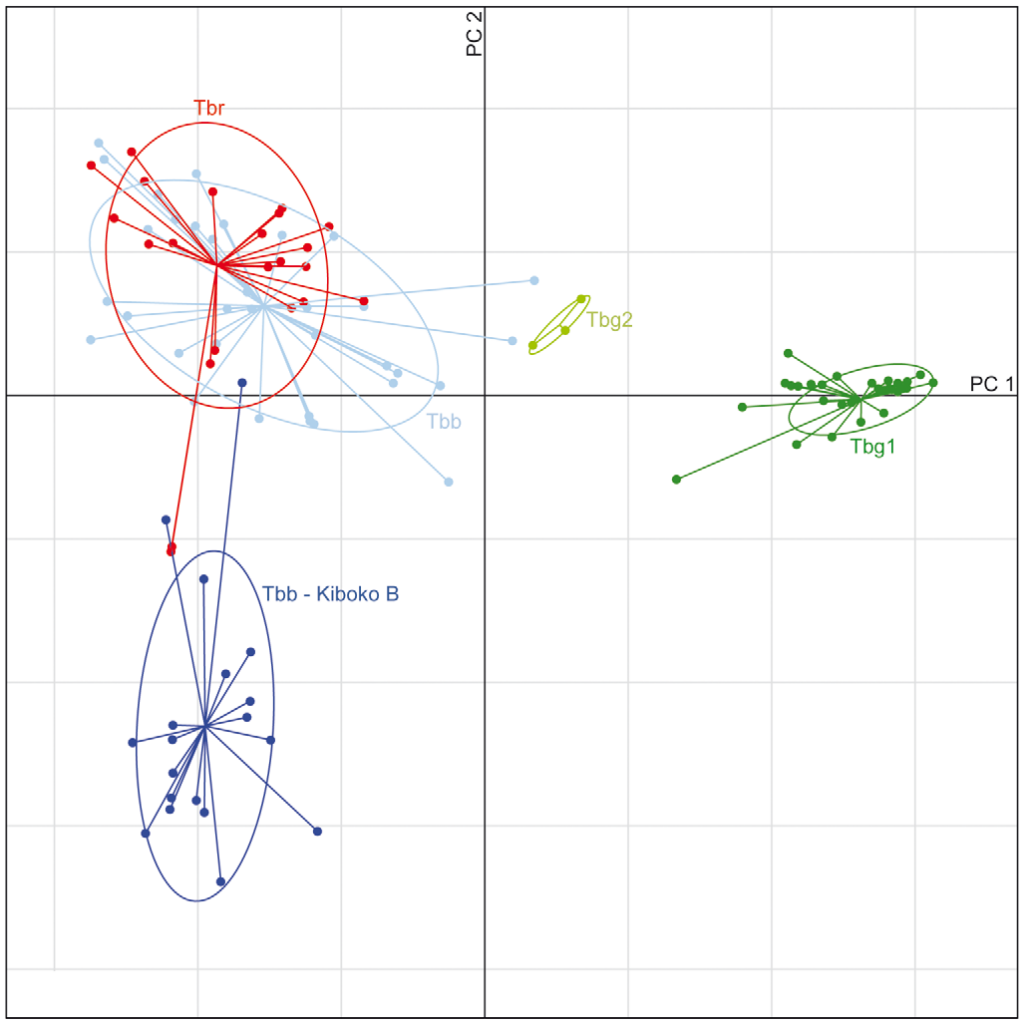
Genetic structure of *Trypanosoma brucei* isolates inferred from principal components analysis. Principal component analysis score plot. Points representing individual genotypes are connected by a line to the centroid of an ellipse, which circumscribes a region encompassing 95% of the variance observed within five trypanosome taxa or subgroups identified by STRUCTURE analysis: *Tbr* (red), *Tbb* Cluster 2 (dark blue), *Tbb* non-Cluster 2 (light blue), *Tbg* group 1 (dark green), *Tbg* group 2 (light green). The first two principal components (PC1 and PC2) explain 31.2% and 8.5% of the total variance in the data, respectively. One sample of *Tbg* group 1 was omitted (b028) due to probable misclassification.

Among isolates of *Tbb* and *Tbr*, only one cluster (Cluster 2, Figure 4) exhibited strong cohesion across various levels of K. This cluster was composed exclusively of *Tbb* from Kenya and Tanzania, and contained all individuals of *Tbb* that had been previously identified as the “Kiboko B” group by isoenzyme and kDNA analysis (Table S1). These isolates also possessed a discrete group of closely related kDNA haplotypes (Hap14–Hap16) that were not shared by any isolates outside of this cluster. The relative differentiation of this group compared to other *Tbb*/*Tbr* and to *Tbg* is visualized in Figure 5, in which the first two axes accounted for 40% of the overall genetic variance. None of the isolates belonging to Cluster 2 tested positive for the SRA gene; however, clustering of isolates at K = 3 and K = 5, as well as the PCA analysis, identified two isolates of *Tbr* (ob065, ob066) that were closely related to isolates in Cluster 2.

Outside of Cluster 2, *Tbb* and *Tbr* exhibited strong genetic similarity as reflected in broadly overlapping 95% ellipses in PCA analysis (Figure 5). At finer scales, clustering of genotypes indicated that in many cases, *Tbb* and *Tbr* isolates are more closely related to each other than they are to other isolates of the same subspecies. Clusters 1, 3, 4, 5, 6, 7, 8 and 9 were each composed of isolates of both *Tbb* and *Tbr* (Figure 4) and in Cluster 8, two isolates of *Tbb* and *Tbr* differed from each other by just one allele at one locus (b179 = RUMP 503 (*Tbb*) and b021 = STIB 391 (*Tbr*)).

We detected the SRA gene in all isolates of *Tbr* except for isolate KETRI 2538, which had been designated as *Tbr* based on isolation from a human patient. Thus, the SRA gene occurred in seven of the ten genetic clusters containing at least one isolate of *Tbb/Tbr* from eastern Africa (Figure 4). Among the samples for which we were able to generate SRA sequence with primers A/E, we detected just two sequence variants. Across the 420 bp of sequence, SRA type 1 was identical to the sequence previously deposited under GenBank accession no. AJ345057 and SRA type 2 was identical to GenBank accession no. AJ345058. These two sequence fragments differed by just three polymorphic sites. SRA type 1 was found in 12 isolates from Uganda, Kenya and Tanzania, while SRA type 2 was found in 11 isolates from Zambia, Ethiopia and Tanzania (Table S1). In the one location where both types occurred sympatrically (Serengeti National Park, Tanzania), we detected isolates in which the two different SRA types associated with the same kDNA lineage (Hap1), but these isolates belonged to different clusters based on their microsatellite genotypes. Across our wider sampling, SRA type 1 was associated with two different kDNA lineages (Hap1 and Hap5), while SRA type 2 was associated with four different lineages (Hap1, Hap4, Hap6 and Hap19; Figure 3 and Figure 4).

Permutation tests indicated that isolates of *Tbb*/*Tbr* found within the same genetic cluster were more likely to originate from the same country than expected by chance alone (p<0.001). This relationship remained significant after excluding individuals from Cluster 2 (p<0.001), i.e. when only those clusters were considered that contained both *Tbb* and *Tbr*. Similarly, individuals from the same genetic cluster were more likely to have been sampled within a similar time period than expected by chance (p<0.001). This, too, remained significant after excluding individuals from Cluster 2, all of which had been isolated between 1970 and 1973 (p = 0.009).

The broad geographical and temporal scale over which samples were collected limited our ability to quantify genetic differences among populations defined by narrow sampling in time and space. Among the groups of isolates that were most cohesive, we observed strong differentiation between isolates from the “Kiboko B” cluster of *Tbb* (Cluster 2) sampled in Tanzania between 1970–1971 and other isolates of *Tbb* sampled in the same place and time (D_EST_ = 0.59±0.11; Table 1). Isolates of *Tbr* sampled concurrently in Tanzania were similarly divergent from the “Kiboko B” cluster (D_EST_ = 0.53±0.12), but exhibited lower differentiation from other *Tbb* (D_EST_ = 0.10±0.06). The low differentiation observed between *Tbr* and *Tbb* (excluding “Kiboko B”) in Tanzania was similar to that observed between isolates of *Tbr* sampled 30 years apart in Uganda (D_EST_ = 0.08±0.08).

**Table 1:** Genetic differentiation between isolates of *Trypanosoma brucei rhodesiense* (*Tbr*) and *T. b. brucei* (*Tbb*).

Index	Taxonomic Group	Country	Years	n	1	2	3	4	5	6	7	8
1	*Tbb*	Tanzania	1970–1971	4	–	0.59	0.10	0.41	0.50	0.77	0.25	0.48
2	*Tbb* “Kiboko B”	Tanzania	1970–1971	14	0.11	–	0.53	0.69	0.75	0.21	0.70	0.75
3	*Tbr*	Tanzania	1970–1971	9	0.06	0.12	–	0.46	0.60	0.59	0.50	0.67
4	*Tbb*	Kenya	1980–1983	4	0.13	0.10	0.11	–	0.31	0.73	0.14	0.41
5	*Tbb*	Zambia	1982–1983	5	0.09	0.04	0.10	0.14	–	0.71	0.55	0.74
6	*Tbb* “Kiboko B”	Kenya	1969–1973	5	0.09	0.11	0.12	0.10	0.12	–	0.83	0.89
7	*Tbr*	Uganda	1960–1961	3	0.18	0.10	0.13	0.17	0.12	0.07	–	0.08
8	*Tbr*	Uganda	1990–1991	4	0.09	0.08	0.13	0.12	0.08	0.05	0.08	–

Country of origin, years of collection and sample size (n) are provided for each group of isolates. Genetic divergence (above diagonal) and standard error (below diagonal) was estimated with eight microsatellite loci using Jost's D.

### Genetic structure - *T. b. gambiense*

Across all levels of partitioning, isolates of *Tbg* group 1 formed a single uniform cluster in STRUCTURE analyses (Cluster 11; Figure 4). Isolates of *Tbg* group 1 also formed a relatively tight and distinct group of genotypes in PCA analysis (Figure 5). Only one isolate identified as *Tbg* group 1 (b028 = STIB 368) did not join this cluster. Within Cluster 11, genetic divergence was low between groups of isolates defined by disease focus and collection date (Table 2). The average pairwise differentiation among all foci was D_EST_ = 0.12. Reflecting this low level of genetic divergence among *Tbg* group 1, we identified just 21 multilocus genotypes among the 54 isolates sampled across central and western Africa. While most of the genotypes that were recovered more than once originated in the same or adjacent countries, two multilocus genotypes were shared between the Ivory Coast and either Equatorial Guinea or the Democratic Republic of Congo (Table S3). One of these multilocus genotypes had persisted for a period of about 18 years (1960–1978), and we identified a second multilocus genotype that had persisted for at least 22 years (1968–1990).

**Table 2:** Genetic divergence between isolates of *Trypanosoma brucei gambiense*.

Index	Taxon	Country	Disease focus	Years	n	1	2	3	4	5	6	7	8
1	*Tbg* group 1	Cameroon	Bipindi	1999	5		0.27	0.07	0.27	0.17	0.19	0.15	0.37
2	*Tbg* group 1	Cameroon	Campo	1996–1999	4	0.17		0.21	−0.05	0.17	0.11	0.08	0.50
3	*Tbg* group 1	Central Afr. Rep.	Batangafo	1999	10	0.06	0.13		0.20	0.12	0.08	0.09	0.42
4	*Tbg* group 1	Eq. Guinea	Mbini	1997	3	0.17	0.03	0.13		0.15	0.10	0.07	0.49
5	*Tbg* group 1	Ivory Coast	-	1978	3	0.11	0.13	0.10	0.13		0.03	0.00	0.28
6	*Tbg* group 1	Sudan	W. Equatoria	2003	5	0.13	0.07	0.12	0.07	0.06		−0.06	0.39
7	*Tbg* group 1	Uganda	Moyo / Omugo	1998–1999	3	0.13	0.06	0.12	0.07	0.06	0.04		0.37
8	*Tbg* group 2	Ivory Coast	-	1978	3	0.15	0.16	0.14	0.16	0.18	0.14	0.15	

Country of origin, focus, years of collection and sample size are provided for each group of isolates. Genetic divergence (above diagonal) and standard error (below diagonal) was estimated with eight microsatellite loci using Jost's D. Negative values of Jost's D may be interpreted as essentially zero differentiation.

Clustering of microsatellite genotypes from isolates identified as *Tbg* group 2, all originating from the Ivory Coast, indicated a close association between these parasites and isolates of *Tbb* and *Tbr* (Figure 4). At K = 11, two *Tbg* group 2 isolates clustered together with isolates of *Tbb* from Uganda, Burkina Faso and Cameroon (Cluster 10). One of these *Tbg* group 2 isolates (b151 = TH02) shared a kDNA haplotype (Hap6) with *Tbb* isolates from Uganda and Tanzania, and *Tbr* isolates from Tanzania and Ethiopia. The other isolate (b032 = STIB386) shared a kDNA haplotype with *Tbb*. The remaining isolate representing *Tbg* group 2 (b146 = TH113) also shared a haplotype (Hap12) with an isolate of *Tbb* (b152 = TSW65, isolated from a pig in the Ivory Coast) and exhibited a signal of mixed ancestry between *Tbg* group 1 (Cluster 11) and *Tbb*/*Tbr* (Cluster 9) based on STRUCTURE analysis (Figure 4). Assignment probabilities for this isolate exhibited 95% credible limits that excluded zero for membership in both Cluster 9 and Cluster 11 (data not shown). The results of Bayesian clustering were reflected in the PCA plot, which placed *Tbg* group 2 genotypes intermediate to *Tbb/Tbr* and *Tbg* group 1 (Figure 5). The two *Tbb* genotypes most closely related to the *Tbg* group 2 cluster derived from Uganda (b009) and Ivory Coast (b152).

## Discussion

Our results, which integrate information from three distinct classes of genetic markers and a broad sampling of trypanosome isolates, corroborate several of the taxonomic and population genetic hypotheses that have emerged over the last decades. Specifically, our data support the lack of monophyly of *Tbr* [14], [33], [37], [69] and highlight the close and reticulated relationships between *Tbr* and *Tbb* [30]. Importantly, we have documented that the SRA gene can occur on genetic backgrounds that encompass most of the diversity found in both *Tbr* and *Tbb*, supporting the proposal that the SRA gene is freely transferable among strains of *T. brucei* in eastern Africa [33]. Furthermore, our results corroborate the low genetic diversity present among isolates of *Tbg* group 1 and confirm the genetic distinction between *Tbg* group 1 and *Tbg* group 2 [70]. Below, we discuss the evolutionary history, taxonomy and genetic structure of *Tbb*/*Tbr* and *Tbg* in more detail.

### *T. b. brucei* and *T. b. rhodesiense*

Human infective trypanosomes from eastern Africa fall into two groups based on clinical characteristics and are characterized by two SRA variants [33], [44]. Our results generally confirm the previously observed geographical partitioning: we found SRA type 1 in Uganda, Kenya and Tanzania, and SRA type 2 in Tanzania, Zambia and Ethiopia. While prior detection of SRA type 2 had been limited to patients sampled in Zambia, Malawi and Ethiopia, we have extended the known range of SRA type 2 to wildlife reservoir hosts in northwest Tanzania. Consequently, Tanzania appears to be a rare location where both SRA types co-occur. Here, trypanosome lineages with SRA type 1 and type 2 were associated with the same kDNA haplotype but distinct microsatellite genotypes. Presuming that an opportunity for dispersal exists, the distinct SRA types may eventually be expected to co-occur elsewhere, raising the potential need for diagnostics that differentiate between these two types.

If the SRA gene, which is responsible for human infectivity of *Tbr* [39], [71], is freely transferable across trypanosome genomes via sexual recombination, then the SRA gene should be associated with trypanosome genetic backgrounds that encompass the diversity observed in *Tbb*. Our results largely corroborate this scenario. We detected SRA in trypanosomes from both of the well-sampled kDNA clades and in seven of the ten genetic clusters inferred from microsatellite-based analysis that contained *Tbb* and/or *Tbr* isolates. Among *Tbb*/*Tbr*, only one cluster of isolates (Cluster 2, Figure 4) appeared to lack SRA while also exhibiting strong differentiation at microsatellite loci. These trypanosomes also possessed a unique group of kDNA haplotypes, potentially indicating that they have not exchanged genes with the other trypanosome lineages represented in our sample. Therefore, this group, containing individuals previously identified as “Kiboko B”, and isolated in the early 1970's from Kenya and Tanzania, may represent true animal-restricted trypanosomes, i.e. *Tbb* [68]. However, this would be a surprising outcome given that at least one cross between the “Kiboko B” group and an unrelated trypanosome lineage (TREU927×STIB386) has been demonstrated in the laboratory [72]. Furthermore, we identified two isolates of *Tbr* that possessed kDNA haplotypes distinct from those possessed by the “Kiboko B” group, but exhibited nuclear genotypes very similar to the “Kiboko B” group (Figure 5). This is consistent with a recombination event between the “Kiboko B” group and an unrelated SRA-positive trypanosome lineage.

Assessed more broadly, our results suggest that SRA has been gained (by recombination) or lost (e.g. by gene conversion) during multiple independent events in the past. For example, Cluster 5 and Cluster 8 are each composed of SRA-positive (type 1) and SRA-negative trypanosomes that are more closely related to each other than they are to trypanosomes in the other cluster. The same is true for Cluster 7 and Cluster 9. Previous work has revealed that human infective and animal-restricted trypanosomes from the same focus showed distinct allele sets, suggesting little recent exchange [18], [19]. On the other hand, our results, which place the results from individual foci in the context of broader geographical sampling, demonstrate that parasites sampled in a restricted time and space often consist of SRA-positive and SRA-negative individuals that may be more closely related to each other than to SRA-positive and SRA-negative parasites recovered from another time and place. In other words, human infective and animal-restricted trypanosomes represent phenotypic variation in a single structured species [73], [74]. Reconciling the apparent lack of interaction between *Tbb* and *Tbr* in a single focus with the capacity for the two to share genes will require more in depth ecological and functional molecular work. Nonetheless, the wide distribution of the SRA gene across trypanosome genotypes has important consequences for the evolution of human infectivity in *Tbb/Tbr* as it suggests that a large amount of genetic diversity is potentially available with which human-infective trypanosomes may eventually respond to selective pressures such as those exerted by drugs. Understanding the time-frame in which SRA can move between trypanosome groups will become particularly important as these genetic groups become better defined with respect to underlying phenotypes of importance, such as drug resistance and disease severity. High throughput next generation sequencing technologies offer the possibility of generating thousands of markers with which to more precisely circumscribe trypanosome groups. Linking these groups to important phenotypes will require large-scale field collections combined with dedicated collaborations with medical staff in disease-endemic countries.

### *T. b. gambiense*

*Tbg* group 1 is the most common form of *Tbg* and is widespread across West and Central Africa. With the exception of one anomalous isolate (STIB368), which is very old (collected in 1959) and may well have been mixed up during prolonged maintenance in the lab, trypanosomes identified as *Tbg* group 1 formed a cohesive genetic group. *Tbg* group 1 genotypes formed a single cluster at all levels of *K* in STRUCTURE analyses, and all isolates shared just two sister haplotypes within clade A of the kDNA phylogeny. Previous studies have used microsatellites to demonstrate limited genetic diversity within the nuclear DNA of *Tbg* group 1 [21], [23], [70]. Our data indicate that this taxon also shows limited diversity in kinetoplast DNA sequence and that extant *Tbg* group 1 kDNA haplotypes fall within a well supported clade representing just a fraction of overall *Tbb/Tbr* diversity. These results suggest that the mechanism governing human infectivity and reproductive isolation of *Tbg* group 1 arose relatively recently. The low virulence in this system is thus not correlated with age of the host-parasite association, as is sometimes suggested based on the wrong assumption [26], [27] that parasites generally evolve towards being more benign as they become better adapted to the host (and vice-versa). The low extant diversity in *Tbg* group 1 may be attributable to a recent and extreme bottleneck. Whatever the underlying cause of the low genetic diversity, the broad distribution of the most common *Tbg* group 1 haplotype across central and western Africa is consistent with the rapid colonization of hosts in this region.

*Tbg* group 2 was originally identified among patient isolates from Ivory Coast; these trypanosomes do not share the low virulence of typical *Tbg* isolates, show variable resistance to the trypanolytic factor in human serum [11] and they do not possess the SRA gene [33],[52],[53]. Identical isolates were recovered from wild and domestic animals in Ivory Coast and Burkina Faso [11]. Relatively few isolates of this type have been recovered, but they have been reported to be genetically heterogeneous [70], distinct from *Tbg* group 1 [11], [75], and closely related to *Tbb* [17], [76]. In our analysis, kDNA haplotypes obtained from *Tbg* group 2 were distinct from haplotypes possessed by isolates of *Tbg* group 1 but fell within a single clade representing all three *T. brucei* subspecies. Each of the three haplotypes possessed by *Tbg* group 2 were shared with isolates classified as *Tbb/Tbr*. Clustering of microsatellite genotypes at *K* = 3 and *K* = 5 also supported a close ancestry between *Tbg* group 2 and *Tbb* or *Tbr*. At *K* = 11 in STRUCTURE analysis, two isolates of *Tbg* group 2 formed a discrete cluster with five isolates of *Tbb*. The remaining isolate exhibited approximately equal probability of membership in *Tbg* group 1 and *Tbb*/*Tbr* Cluster 9, supporting a hybrid origin for some members of *Tbg* group 2. Although many of the associations above point to close relationships between isolates of *Tbg* group 2 from western Africa and isolates of *Tbb* or *Tbr* originating in eastern Africa, these results are likely biased by a lack of sampling of *Tbb* in central and western Africa. Future sampling and genotyping of *Tbb* in these regions should help to resolve the evolutionary origins of human infectivity in the *gambiense* group of trypanosomes.

## Supporting Information

Table S1 Taxonomic and collection data for isolates of *Trypanosoma brucei* used for microsatellite (n = 140) and CO1 (n = 87) analyses, sorted by taxon.
(0.47 MB DOC)

Table S2 Isolates of *Trypanosoma brucei brucei* (italics), *T. b. rhodesiense* (bold), *T. b. gambiense* group 1 (plain font), and *T. b. gambiense* group 2 (underlined) sharing the same haplotype, based on partial COI sequences.
(0.09 MB DOC)

Table S3 Isolate codes, taxonomic classification, countries of origin, and dates of collection for groups of isolates with identical genotypes.
(0.11 MB DOC)
