# Supplementary material for: Phylogeography and Taxonomy of Trypanosoma brucei
Source: PLoS Negl Trop Dis. 2011 Feb 8;5(2):e961. doi: 10.1371/journal.pntd.0000961 (PMC3035665; doi:10.1371/journal.pntd.0000961)
Supplement: Table S1 — Taxonomic and collection data for isolates of Trypanosoma brucei used for microsatellite (n = 140) and CO1 (n = 87) analyses, sorted by taxon. (0.47 MB DOC) [file pntd.0000961.s001.doc]

Table S1. Taxonomic and collection data for isolates of *Trypanosoma brucei* used for microsatellite (n = 140) and CO1 (n = 87) analyses, sorted by taxon.

| **Taxon^a^** | **Isolate code** | **Haplotype^b^** | **Cluster^c^** | **SRA^d^** | **Isoenzyme^e^** | **kDNA^f^** | **Country^g^** | **Full isolate name** | **Origin and synonymous isolate names^h^** | **Host^i^** | **Year^j^** |
| --- | --- | --- | --- | --- | --- | --- | --- | --- | --- | --- | --- |
| Tbb | c003 | Hap14 | 2 | - ^(3)^ |  | Kiboko B | K | 927/4 | Kiboko | *G. pall.* | 1970 |
| Tbb | c004 | Hap1 | 9 | - ^(1)^ |  |  | U | EATRO 1296 | Lugala | *G. pall.* | 1969 |
| Tbb | c005 | Hap5 | 5 | - ^(3)^ | Busoga^(1)^ |  | K | Fly 409 clone 21 | Lambwe valley | *G. pall.* | 1983 |
| Tbb | c006 | Hap18 | 3 | - ^(1)^ | Kakumbi^(1)^ | Kiboko A | Z | H3 | Luangwa valley | lion | 1974 |
| Tbb | c007 | Hap18 | 3 | - ^(1,2)^ | Kakumbi^(1)^ | Kiboko A | Z | J10 | Luangwa valley | hyaena | 1973 |
| Tbb | c008 | Hap5 | 9 | - ^(1)^ |  |  | U | Katerema 41 |  | cow | 1990 |
| Tbb | c009 | Hap15 | 2 | - ^(1,3)^ | Kiboko^(1)^ | Kiboko B | K | KETRI 1738 | Kiboko | sheep | 1970 |
| Tbb | c012 | Hap15 | 2 | - ^(3)^ | Kiboko^(1)^ | Kiboko B | K | KETRI 2090 | Meru | cow | 1973 |
| Tbb | c013 | Hap16 | 2 | - ^(3)^ | Kiboko^(1)^ | Kiboko B | K | KETRI 2108 | Meru | cow | 1973 |
| Tbb | c014 | Hap13 | 5 | - ^(1)^ | Busoga^(1)^ | Sindo | K | LF1 | Lambwe valley | *G. pall.* | 1980 |
| Tbb | c015 | Hap13 | 5 | - ^(1)^ | Kiboko^(1)^ | Sindo | K | LUMP 1342 (LUMP 450) | Sindo, derived from MRC442, fly S31 | *G. pall.* | 1969 |
| Tbb | c016 | Hap14 | 2 | - ^(1,2)^ | Kiboko^(1)^ | Kiboko B | K | LUMP 266 | Kiboko, derived from MRC 241, fly K4 | *G. pall.* | 1969 |
| Tbb | c017 | Hap5 | 5 | - ^(3)^ |  |  | K | LVBG 118N | Lambwe valley | cow | 1980 |
| Tbb | c018 | Hap5 | -- | - ^(1)^ |  | West | K | LVBG 3N | Lambwe valley | cow | 1980 |
| Tbb | c019 | Hap3 | 4 | - ^(1,2)^ |  | East | K | M249 | Matuga | sheep | 1981 |
| Tbb | c020 | Hap5 | 5 | - ^(3)^ | Busoga^(1)^ | West | K | RB67 | Lambwe valley | reedbuck | 1970 |
| Tbb | c022 | Hap17 | -- | - ^(1)^ | Kiboko^(1)^ | Kiboko B | T | STIB 215 | ex lion 245 (SNP) | lion | 1971 |
| Tbb | c023 | Hap1 | 7 | - ^(3)^ |  |  | T | STIB 221 | ex kongoni 258 (SNP) | kongoni | 1971 |
| Tbb | c024 | Hap13 | 4 | - ^(3)^ | Sindo^(1)^ |  | Z | TRPZ 239 | Luangwa valley | giraffe | 1982 |
| Tbb | c026 | Hap19 | 3 | - ^(3)^ | Kakumbi^(1)^ | Kiboko A | Z | TRPZ 286 (pop 1) | Luangwa valley | *G. pall.* | 1983 |
| Tbb | c027 | Hap2 | 3 | - ^(3)^ | Kakumbi^(1)^ | Mixed | Z | TRPZ 317 (clone 4) | Luangwa valley | *G. mors.* | 1983 |
| Tbb | c028 | Hap13 | 4 | - ^(1)^ | Sindo^(1)^ | Sindo | Z | TRPZ 320 (pop 1) | Luangwa valley | *G. pall.* | 1983 |
| Tbb | c029 | Hap13 | 4 | - ^(3)^ | Sindo^(1)^ |  | Z | TRPZ 323 | Luangwa valley | *G. mors.* | 1983 |
| Tbb | b009 | Hap10 | 10 | - ^(3)^ |  |  | U | STIB 777AE | Busoga | *G. fusc.* | 1971 |
| Tbb | b010 | -- | 6 | - ^(2,3)^ |  |  | T | STIB 920 | ex kongoni 263 / STIB 246/348 | kongoni | 1971 |
| Tbb | b013 | -- | 2 | - ^(3)^ |  |  | T | STIB 201 | ex lion 201 (SNP) | lion | 1971 |
| Tbb | b014 | -- | 2 | - ^(3)^ |  |  | T | STIB 213 | ex hyaena 228 (SNP) | hyaena | 1971 |
| Tbb | b030 | -- | 5 | - ^(3)^ |  |  | U | STIB 776 | Busoga; KETRI 1956 | *G. fusc.* | 1971 |
| Tbb | b031 | -- | 4 | - ^(3)^ |  |  | O | STIB 794 A | CP 547/ Somalia | cattle | 1985 |
| Tbb | b051 | -- | 3 | - ^(3)^ |  |  | U | STIB 340 | EATRO (received from Tororo) | hippo | 1961 |
| Tbb | b052 | -- | 2 | - ^(3)^ |  |  | K | EATRO 1532 | Kiboko MRC | n/a | n/a |
| Tbb | b055 | -- | 7 | - ^(3)^ |  |  | T | STIB 337 | ex hyaena 284 (SNP) / STIB 240 | hyaena | 1971 |
| Tbb | b058 | -- | 2 | - ^(3)^ |  |  | U | STIB 390 | EATRO 1856; SNP ex kongoni 49 | kongoni | 1970 |
| Tbb | b059 | -- | 2 | - ^(3)^ |  |  | T | STIB 205 | ex lion 207 (SNP) | lion | 1971 |
| Tbb | b061 | -- | 2 | - ^(3)^ |  |  | T | STIB 217 | ex lion 247 (SNP) | lion | 1971 |
| Tbb | b062 | -- | 2 | - ^(3)^ |  |  | T | STIB 218 | ex lion 244 (SNP) | lion | 1971 |
| Tbb | b063 | -- | 2 | - ^(3)^ |  |  | T | STIB 236 | ex lion 275 (SNP) | lion | 1971 |
| Tbb | b067 | -- | 2 | - ^(3)^ |  |  | T | STIB 202 | ex lion 218 (SNP) | lion | 1971 |
| Tbb | b068 | -- | 2 | - ^(3)^ |  |  | T | STIB 204 | ex lion 203 (SNP) | lion | 1971 |
| Tbb | b069 | -- | 2 | - ^(3)^ |  |  | T | STIB 207 | ex lion 210 (SNP) | lion | 1971 |
| Tbb | b070 | -- | 2 | - ^(3)^ |  |  | T | STIB 209 | ex lion 204 (SNP) | lion | 1971 |
| Tbb | b071 | -- | 2 | - ^(3)^ |  |  | T | STIB 210 | ex lion 205 (SNP) | lion | 1971 |
| Tbb | b075 | -- | 2 | - ^(3)^ |  |  | T | STIB 216 | ex hyaena 248 (SNP) | hyaena | 1971 |
| Tbb | b076 | -- | 2 | - ^(3)^ |  |  | T | STIB 219 | ex lion 241 (SNP) | lion | 1971 |
| Tbb | b086 | Hap6 | 5 | - ^(2)^ |  |  | U | AnTat 1.1 | EATRO 1125 – LUMP581 | bushbuck | 1966 |
| Tbb | b087 | Hap6 | 5 | - ^(3)^ |  |  | T | STIB 247 | ex kongoni 263 (SNP) | kongoni | 1971 |
| Tbb | b088 | Hap3 | 6 | - ^(3)^ |  |  | K | STIB 345 AB | EATRO/MRC 1529, Kiboko | *G. pall.* | 1969 |
| Tbb | b089 | Hap1 | 1 | - ^(3)^ |  |  | T | STIB 366 Clone1 | S42/030 | warthog | 1966 |
| Tbb | b091 | Hap1 | 9 | - ^(3)^ |  |  | U | STIB 783 | EATRO 1244 (LUMP1026) | *G. pall.* | 1969 |
| Tbb | b113 | Hap11 | 10 | - ^(3)^ |  |  | F | GAOUA 89 | Gaoua | ox | 1989 |
| Tbb^k^ | b152 | Hap12 | 9 | - ^(3)^ |  |  | I | TSW 65 (KP1) | Disease focus Vavoua | pig | 1982 |
| Tbb^k^ | b153 | Hap7 | 10 | - ^(3)^ | 19^(2)^ |  | C | P16F | Disease focus Fontem | pig | 1999 |
| Tbb^k^ | b154 | Hap7 | 10 | - ^(3)^ |  |  | C | P7F | Disease focus Fontem | pig | 1999 |
| Tbb^k^ | b155 | Hap7 | 10 | - ^(3)^ | 19^(2)^ |  | C | P8F | Disease focus Fontem | pig | 1999 |
| Tbb | b178 | Hap1 | 9 | - ^(3)^ |  |  | T | RUMP501 |  | cf. cattle | 1956 |
| Tbb | b179 | Hap5 | 8 | - ^(1)^ |  |  | K | RUMP503 | Central Nyanza, Alego/ EATRO 795/ LUMP 227 | ox | 1965 |
| Tbb | b185 | -- | 9 | - ^(3)^ |  |  | U | STIB 795 | S 427.1 ILRAD (from S. Black), SE Uganda | *G. pall.* | 1960 |
| Tbr | c002 | Hap1 | 7 | 2 ^(3)^ |  |  | T | STIB 250 | ex kongoni 257 (SNP) / STIB229 | kongoni | 1971 |
| Tbr | c025 | Hap19 | 3 | 2 ^(3)^ |  |  | Z | TRPZ 260 | Luangwa valley | ox | 1982 |
| Tbr | b006 | Hap1 | 8 | + ^(3)^ |  |  | U | STIB 799 | ex man after 4th relapse; Busoga; EATRO 243 | man | 1961 |
| Tbr | b012 | Hap6 | 9 | 2 ^(3)^ |  |  | T | STIB 056 | ex waterbuck 82/ EATRO 1836 | waterbuck | 1971 |
| Tbr | b017 | -- | 5 | + ^(3)^ |  |  | K | STIB 365 | ex man (1st stage); Homa Bay | man | 1973 |
| Tbr | b018 | -- | 4 | + ^(3)^ |  |  | B | STIB 338 | Mababe, Ngamiland/Dunnels-strain | man | 1960 |
| Tbr | b021 | Hap5 | 8 | 1 ^(3)^ |  |  | U | STIB 391 | Lugala/ ETat 10/ILRAD 853/ TREU 164 | *G. pall.* | 1960 |
| Tbr | b022 | -- | 6 | 1 ^(3)^ |  |  | T | STIB 703-A | male 15y/ CSF/ Ifakara | man | 1982 |
| Tbr | b024 | -- | 9 | 1 ^(3)^ |  |  | K | STIB 706 | Lambwe Valley/ LVH-74 | man | 1980 |
| Tbr | b025 | -- | 3 | - ^(3)^ |  |  | M | KETRI 2538 | Tete Province | man | 1980 |
| Tbr | b026 | -- | 9 | 2 ^(3)^ |  |  | E | STIB 707 | Illubabor, EATRO 1713 | man | 1970 |
| Tbr | b027 | -- | 5 | 1 ^(3)^ |  |  | U | EATRO 0240 | 2nd relapse; Busoga | man | 1961 |
| Tbr | b053 | -- | 7 | 2 ^(3)^ |  |  | T | STIB 324 | ex hyaena 282/290 (SNP) / STIB 235 | hyaena | 1971 |
| Tbr | b054 | Hap1 | 7 | 2 ^(3)^ |  |  | T | STIB 316 | ex lion 236 (SNP) / STIB 236 | lion | 1971 |
| Tbr | b056 | -- | 7 | 2 ^(3)^ |  |  | T | STIB 286 | ex hyaena 293 (SNP) / STIB 243 | hyaena | 1971 |
| Tbr | b057 | -- | 7 | 2 ^(3)^ |  |  | T | STIB 243 | ex hyaena 293 (SNP) | hyaena | 1971 |
| Tbr | b065 | Hap1 | 1 | 1 ^(3)^ |  |  | T | STIB 262 | ex kongoni 47 (SNP) / EATRO 1873 | kongoni | 1970 |
| Tbr | b066 | Hap1 | 1 | 1 ^(3)^ |  |  | T | STIB 263 | ex kongoni 47 (SNP) / EATRO 1873 | kongoni | 1970 |
| Tbr | b078 | -- | 5 | 1 ^(3)^ |  |  | K | EATRO 0237 | isolate from the 3rd relapse; Bungala | man | 1961 |
| Tbr | b093 | Hap1 | 7 | 2 ^(3)^ |  |  | T | STIB 364-A | ex lion 278 (SNP), STIB241 ex volunteer | lion | 1971 |
| Tbr | b094 | -- | 6 | 1 ^(3)^ |  |  | T | STIB 704 | Ifakara | man | 1982 |
| Tbr | b095 | Hap6 | 9 | 2 ^(3)^ |  |  | E | STIB 809 | Illubabor, EATRO 1192, Gambella 1 | man | 1967 |
| Tbr | b096 | Hap5 | 8 | 1 ^(2,3)^ |  |  | U | STIB 848 | UTRO 030790 | man | 1990 |
| Tbr | b097 | -- | 8 | 1 ^(2,3)^ |  |  | U | STIB 849 | UTRO 150291 | man | 1991 |
| Tbr | b098 | -- | 8 | 1 ^(2,3)^ |  |  | U | STIB 851 | UTRO 120890A | man | 1990 |
| Tbr | b099 | Hap5 | 8 | 1 ^(3)^ |  |  | U | STIB 854 | UTRO 281290 | man | 1990 |
| Tbr | b156 | Hap4 | 6 | 2 ^(3)^ | 18^(2)^ |  | Z | TRPZ 166 | Kakumbi | ox | 1982 |
| Tbg 1 | b007 | Hap8 | 11 |  |  |  | I | STIB 930 | Vavoua, TH-1/ 78E (031); STIB 754 | man | 1978 |
| Tbg 1 | b028 | -- | 6 | - ^(3)^ |  |  | U | STIB 368 | West-Nile 1959/ EATRO 210 | man | 1959 |
| Tbg 1 | b033 | -- | 11 |  |  |  | I | STIB 733 | Vavoua/ TH-1/78/(020) | man | 1978 |
| Tbg 1 | b034 | -- | 11 |  |  |  | L | STIB 756 | TH-11/81 'G4-3'; Gongota | man | 1981 |
| Tbg 1 | b048 | Hap8 | 11 | - ^(3)^ |  |  | A | 001 K1 Angola |  | man | 1998 |
| Tbg 1 | b079 | -- | 11 | - ^(3)^ |  |  | I | DAL 1086 | Daloa, 229 days in mouse | man | 1987 |
| Tbg 1 | b080 | -- | 11 |  |  |  | I | DAL 1086 R | Daloa, DAL1086 relapse after 4 mths. 59 d in mouse | man | 1987 |
| Tbg 1 | b082 | -- | 11 |  |  |  | I | DAL 1402 | Daloa | man | 1990 |
| Tbg 1 | b083 | -- | 11 |  |  |  | I | STIB 717 | Daloa, TH-Dal 069 | man | cf. 1978 |
| Tbg 1 | b102 | -- | 11 |  |  |  | I | STIB 755 | TH-2/78E (031) | man | 1978 |
| Tbg 1 | b107 | Hap8 | 11 |  |  |  | S | K00014JD | Western Equatoria, Lazoh, Midi | man | 2003 |
| Tbg 1 | b109 | -- | 11 |  |  |  | S | K0303030 | Western Equatoria, Kotobi, Goribalan | man | 2003 |
| Tbg 1 | b110 | -- | 11 |  |  |  | S | K0303043 | Western Equatoria, Wiri Lui | man | 2003 |
| Tbg 1 | b111 | -- | 11 |  |  |  | S | K0303045 | Western Equatoria, Nyau 1 | man | 2003 |
| Tbg 1 | b112 | -- | 11 |  |  |  | S | K0303048 | Western Equatoria, Lazoh, Buangyi | man | 2003 |
| Tbg 1 | b115 | Hap8 | 11 |  | 1^(2)^ |  | C | A005 | Disease focus Fontem | man | 1988 |
| Tbg 1 | b116 | Hap8 | 11 |  |  |  | C | BIP 04 | Disease focus Bipindi | man | 1999 |
| Tbg 1 | b117 | Hap8 | 11 |  |  |  | C | BIP 08 | Disease focus Bipindi | man | 1999 |
| Tbg 1 | b118 | Hap8 | 11 |  |  |  | C | BIP 09 | Disease focus Bipindi | man | 1999 |
| Tbg 1 | b119 | -- | 11 |  |  |  | C | BIP 40 | Disease focus Bipindi | man | 1999 |
| Tbg 1 | b120 | -- | 11 |  |  |  | C | BIP 42 | Disease focus Bipindi | man | 1999 |
| Tbg 1 | b121 | Hap8 | 11 |  |  |  | C | C 3359 | Disease focus Campo | man | 1998 |
| Tbg 1 | b122 | Hap8 | 11 |  |  |  | C | DOUME1 | Disease focus Doume | man | 2000 |
| Tbg 1 | b124 | Hap8 | 11 |  | 1^(2)^ |  | C | P26F | Disease focus Fontem | man | 1999 |
| Tbg 1 | b125 | Hap8 | 11 |  |  |  | C | SEMI | Disease focus Campo | man | 1996 |
| Tbg 1 | b126 | Hap9 | 11 |  |  |  | C | SOMABc | Disease focus Campo | man | 1999 |
| Tbg 1 | b127 | Hap8 | 11 |  |  |  | C | TSEMESO | Disease focus Campo | man | 1998 |
| Tbg 1 | b128 | Hap8 | 11 |  |  |  | R | BAT 10 | Disease focus Batangafo | man | 1999 |
| Tbg 1 | b129 | -- | 11 |  |  |  | R | BAT 31 | Disease focus Batangafo | man | 1999 |
| Tbg 1 | b130 | Hap8 | 11 |  |  |  | R | BAT 37 | Disease focus Batangafo | man | 1999 |
| Tbg 1 | b131 | Hap8 | 11 |  |  |  | R | BAT 39 | Disease focus Batangafo | man | 1999 |
| Tbg 1 | b132 | Hap8 | 11 |  |  |  | R | BAT 40 | Disease focus Batangafo | man | 1999 |
| Tbg 1 | b133 | Hap8 | 11 |  |  |  | R | BAT 42 | Disease focus Batangafo | man | 1999 |
| Tbg 1 | b134 | Hap8 | 11 |  |  |  | R | BAT 45 | Disease focus Batangafo | man | 1999 |
| Tbg 1 | b135 | Hap8 | 11 |  |  |  | R | BAT 51 | Disease focus Batangafo | man | 1999 |
| Tbg 1 | b136 | Hap8 | 11 |  |  |  | R | BAT 58 | Disease focus Batangafo | man | 1999 |
| Tbg 1 | b137 | Hap8 | 11 |  |  |  | R | BAT 60 | Disease focus Batangafo | man | 1999 |
| Tbg 1 | b138 | Hap8 | 11 |  | 4^(3)^ |  | R | BIBIANA | Disease focus Obo | man | 1999 |
| Tbg 1 | b139 | -- | 11 |  | 4^(3)^ |  | R | MBADI | Disease focus Obo | man | 1999 |
| Tbg 1 | b140 | Hap8 | 11 |  | 3^(3)^ |  | H | NATONDJI | Disease focus Moundou | man | 1998 |
| Tbg 1 | b141 | Hap8 | 11 |  |  |  | N | DEMBA | Disease focus Bouenza | man | 1989 |
| Tbg 1 | b142 | Hap8 | 11 |  | 1^(3)^ |  | N | MALOUNDA | Disease focus Bouenza | man | 1989 |
| Tbg 1 | b143 | Hap8 | 11 |  | 1^(3)^ |  | Q | 13.97D | Disease focus Mbini | man | 1997 |
| Tbg 1 | b144 | -- | 11 |  | 1^(3)^ |  | Q | 14.97D | Disease focus Mbini | man | 1997 |
| Tbg 1 | b145 | Hap8 | 11 |  | 5^(3)^ |  | Q | 15.97D | Disease focus Mbini | man | 1997 |
| Tbg 1 | b147 | Hap8 | 11 |  |  |  | U | F43UG | Disease focus Omugo | man | 1999 |
| Tbg 1 | b148 | Hap8 | 11 |  |  |  | U | R56UG | Disease focus Moyo | man | 1998 |
| Tbg 1 | b149 | -- | 11 |  |  |  | U | R60UG | Disease focus Moyo | man | 1998 |
| Tbg 1 | b186 | Hap8 | 11 |  |  |  | D | ITMAP020578 | Bandundu/Lebu. Stock "Ntuma". | man | 1977 |
| Tbg 1 | b187 | Hap8 | 11 |  |  |  | D | ITMAP141267 | Bandundu/Lac Mai ndombe. Stock "Patowsky" | man | 1960 |
| Tbg 1 | b188 | -- | 11 |  |  |  | D | ITMAP160986 | Kinshasa. Stock "Bosendja" | man | 1972 |
| Tbg 1 | b189 | Hap8 | 11 |  |  |  | D | ITMAP1780 | cf ITMAP281277. Bandundu/Lebu. Stock "Ngaki" | man | 1977 |
| Tbg 1 | b190 | -- | 11 |  |  |  | D | ITMAP211290 | Kimpese Sud. Stock "Dieyi" | man | 1990 |
| Tbg 1 | b191 | Hap8 | 11 |  |  |  | D | ITMAS060401 | Bas Congo/Mongo-Bemba. Stock "Logra" | man | 1968 |
| Tbg 1 | b202 | Hap8 | 11 |  |  |  | D | ITMAP210879 | Bas Congo/Kwilu-Ngongo. Stock "Moer82" | man | 1970 |
| Tbg 2 | b032 | Hap10 | 10 |  |  |  | I | STIB 386 | TH-114/ 78E(020) | man | 1978 |
| Tbg 2 | b146 | Hap12 | 11 |  |  |  | I | TH 113 | MHOM/CI/78/TH113 | man | 1978 |
| Tbg 2 | b151 | Hap6 | 10 |  | 8^(2)^ |  | I | TH 02 | MHOM/CI/78/TH2 | man | 1978 |

^a^: Tbb, *T. b. brucei*; Tbg 1, *T. b. gambiense* group 1; Tbg 2, *T. b. gambiense* group 2; Tbr, *T. b. rhodesiense*

^b^: Haplotype code corresponds to labels in Figures 2 and 3; --, not determined

^c^: Cluster number corresponds to results of STRUCTURE analysis with K = 11 (see Figure 4); --, not determined

^d^: Information on presence (+) or absence (-) of SRA and on SRA type where known (1, SRA type 1; 2, SRA type 2) aggregated from Gibson et al. [1] (1) , Radwanska et al. [2] (2) and this study (3)

^e^: isoenzyme group from Godfrey et al. [3] and [4] (1), [5] (2), or [6] (3)

^f^: kDNA maxicircle type

^g^: A, Angola; B, Botswana; C, Cameroon; D, Democratic Republic of Congo; E, Ethiopia; F, Burkina Faso; H, Chad; I, Ivory Coast; K, Kenya; L, Liberia; M, Mozambique; N, Congo Brazzaville; O, Somalia; Q, Equatorial Guinea; R, Central African Republic; S, Sudan; T, Tanzania; U, Uganda; Z, Zambia

^h^: SNP, Serengeti National Park, Tanzania

^i^: *G. pall.*, *Glossina pallidipes*; *G. mors.*, *G. morsitans*; *G. fusc.*, *G. fuscipes*

^j^: year of isolation

^k^: listed as „*T. brucei* non-gambiense group 1“ in [5]

**References**

1. Gibson WC, Backhouse T, Griffiths A (2002) The human serum resistance associated gene is ubiquitous and conserved in *Trypanosoma brucei rhodesiense* throughout East Africa. Infection, Genetics and Evolution 1: 207-214.

2. Radwanska M, Chamekh M, Vanhamme L, Claes F, Magez S, et al. (2002) The serum resistance-associated gene as a diagnostic tool for the detection of *Trypanosoma brucei rhodesiense*. American Journal of Tropical Medicine and Hygiene 67: 684-690.

3. Godfrey DG, Baker RD, Rickman LR, Mehlitz D (1990) The distribution, relationships and identification of enzymic variants within the subgenus *Trypanozoon*. Advances in Parasitology 29: 1-74.

4. Stevens JR (1991) Numerical studies on the biochemical characteristics of trypanosomes in the subgenus *Trypanozoon*. Bristol, UK: University of Bristol.

5. Nkinin SW, Njiokou F, Penchenier L, Grebaut P, Simo G, et al. (2002) Characterization of Trypanosoma brucei s.l. subspecies by isoenzymes in domestic pigs from the Fontem sleeping sickness focus of Cameroon. Acta Tropica 81: 225-232.

6. Nkinin SW, Njiokou F, Grebaut P, Penchenier L, Bureau P, et al. (1999) Isoenzyme characterization of *Trypanosoma brucei* s.l. stocks from different foci in the Central African Region. Bull liais doc OCEAC 32: 9-16.
