# Supplementary material for: Phylogeography and Taxonomy of Trypanosoma brucei
Source: PLoS Negl Trop Dis. 2011 Feb 8;5(2):e961. doi: 10.1371/journal.pntd.0000961 (PMC3035665; doi:10.1371/journal.pntd.0000961)
Supplement: Table S2 — Isolates of Trypanosoma brucei brucei (italics), T. b. rhodesiense (bold), T. b. gambiense group 1 (plain font), and T. b. gambiense group 2 (underlined) sharing the same haplotype, based on partial COI sequences. (0.09 MB DOC) [file pntd.0000961.s002.doc]

Table S2. Isolates of *Trypanosoma brucei brucei* (italics), *T. b. rhodesiense* (bold), *T. b. gambiense* group 1 (plain font), and *T. b. gambiense* group 2 (underlined) sharing the same haplotype, based on partial COI sequences.

| **Haplotype** | **Isolate codes** |
| --- | --- |
| Hap1 | **c002**, *c004*, *c023*, **b006**, **b054**, **b065**, **b066**, *b089*, *b091*, **b093**, *b178* |
| Hap2 | *c027* |
| Hap3 | *c019*, *b088* |
| Hap4 | **b156** |
| Hap5 | *c005*, *c008*, *c017*, *c018*, *c020*, **b021**, **b096**, **b099**, *b179* |
| Hap6 | **b012**, *b086*, *b087*, **b095**, b151 |
| Hap7 | *b153, b154, b155* |
| Hap8 | b007, b048, b107, b115, b116, b117, b118, b121, b122, b124, b125, b127, b128, b130, b131, b132, b133, b134, b135, b136, b137, b138, b140, b141, b142, b143, b145, b147, b148, b186, b187, b189, b191, b202 |
| Hap9 | b126 |
| Hap10 | *b009*, b032 |
| Hap11 | *b113* |
| Hap12 | b146, *b152* |
| Hap13 | *c014*, *c015*, *c024*, *c028*, *c029* |
| Hap14 | *c003*, *c016* |
| Hap15 | *c009*, *c012* |
| Hap16 | *c013* |
| Hap17 | *c022* |
| Hap18 | *c006*, *c007* |
| Hap19 | **c025**, *c026* |

See Table S1 for isolate details.
