# Supplementary material for: Phylogeography and Taxonomy of Trypanosoma brucei
Source: PLoS Negl Trop Dis. 2011 Feb 8;5(2):e961. doi: 10.1371/journal.pntd.0000961 (PMC3035665; doi:10.1371/journal.pntd.0000961)
Supplement: Table S3 — Isolate codes, taxonomic classification, countries of origin, and dates of collection for groups of isolates with identical genotypes. (0.11 MB DOC) [file pntd.0000961.s003.doc]

Table S3. Isolate codes, taxonomic classification, countries of origin, and dates of collection for groups of isolates with identical genotypes.

| **Isolate codes** | **Taxon** | **Countries of origin** | **Dates of collection** |
| --- | --- | --- | --- |
| c004, b091 | *T. b. brucei* | Uganda | 1969 |
| b178, b185 | *T. b. brucei* | Tanzania, Uganda | 1956, 1970 |
| b059, b071 | *T. b. brucei* | Tanzania | 1971 |
| b053, b054, b093, c002 | *T. b. rhodesiense* | Tanzania | 1971 |
| b056, b057 | *T. b. rhodesiense* | Tanzania | 1971 |
| b012, b095 | *T. b. rhodesiense* | Tanzania, Ethiopia | 1971, 1968 |
| b022, b094 | *T. b. rhodesiense* | Tanzania | 1982 |
| b191, b202, b190 | *T. b. gambiense* group 1 | Democratic Republic of Congo | 1968, 1970, 1990 |
| b033, b187, b188 | *T. b. gambiense* group 1 | Ivory Coast, Democratic Republic of Congo | 1978, 1960, 1972 |
| b186, b189 | *T. b. gambiense* group 1 | Democratic Republic of Congo | 1977 |
| b034, b082 | *T. b. gambiense* group 1 | Liberia, Ivory Coast | 1981, 1990 |
| b141, b128, b134 | *T. b. gambiense* group 1 | Congo, Central African Republic | 1989, 1999 |
| b007, b083, b102 | *T. b. gambiense* group 1 | Ivory Coast | 1978 |
| b107, b111, b112, b148 | *T. b. gambiense* group 1 | Sudan, Uganda | 2003, 1998 |
| b121, b125-b127, b143-b145 | *T. b. gambiense* group 1 | Congo, Equatorial Guinea, Cameroon | 1996-1999 |
| b116, b117, b118, b119 | *T. b. gambiense* group 1 | Cameroon | 1999 |
| b129-b133, b135-b137 | *T. b. gambiense* group 1 | Central African Republic | 1999 |
| b142, b138, b139 | *T. b. gambiense* group 1 | Congo, Central African Republic | 1989, 1999 |
| b147, b149 | *T. b. gambiense* group 1 | Uganda | 1998, 1999 |
| b115, b122 | *T. b. gambiense* group 1 | Cameroon | 1988, 2000 |

See Table S1 for isolate details.
